# Racial Disparities in Flavored Tobacco Product Use, Curiosity, Susceptibility, and Harm Perception, National Youth Tobacco Survey 2019–2020

**DOI:** 10.1089/heq.2022.0087

**Published:** 2023-02-28

**Authors:** Christina Vaughan Watson, Samantha Puvanesarajah, Nikki A. Hawkins, Katrina F. Trivers

**Affiliations:** Office on Smoking and Health, National Center for Chronic Disease Prevention and Health Promotion, CDC, Atlanta, Georgia, USA.

**Keywords:** tobacco, vaping, menthol, disparities, youth

## Abstract

**Introduction:**

Studies characterizing differences in youth flavored tobacco product use prevalence, curiosity/susceptibility, and harm perceptions by race and ethnicity are limited. This study comprehensively examines flavored tobacco product use and harm perceptions among U.S. middle and high school students, by race and ethnicity.

**Methods:**

Data came from the 2019 (*N*=19,018) and 2020 (*N*=14,531) National Youth Tobacco Surveys (NYTS). Weighted prevalence estimates of flavored tobacco product use and curiosity, susceptibility, and harm perception are reported by race and ethnicity (non-Hispanic [NH] White, NH Black, Hispanic, or NH Other). *t*-Tests assessed differences in prevalence by years and racial/ethnic groups.

**Results:**

Among youth with past 30-day tobacco use, use of most flavored tobacco products increased across all racial/ethnic groups; the largest increase was observed among Hispanic youth using other flavored tobacco products (30.3%). The group with the highest susceptibility to future electronic cigarette (e-cigarette) use was Hispanic students (42.3%). Hispanic students had the highest curiosity about and susceptibility to future use of cigarettes and cigars as well.

**Conclusions:**

Increases in the use of and higher susceptibility to other flavored tobacco products, particularly among Hispanic youth, suggest a need for additional changes in environmental conditions and possibly targeted or tailored tobacco control interventions for Hispanic youth.

**Implications:**

Given that flavored tobacco use is prevalent among youth and aggressively marketed more to racial/ethnic minority populations, it is important to understand how susceptibility and perceptions relate to tobacco use. Our results suggest a need for a better understanding of social and environmental factors that drive tobacco use behaviors and perceptions, particularly among Hispanic youth, to address the root causes of these differences and create more equitable tobacco control interventions.

## Introduction

The tobacco product landscape continues to evolve rapidly with the introduction of new products and changing regulations. In 2020, electronic cigarettes (e-cigarettes) were the most common tobacco product used by middle and high school students,^1^ and flavored e-cigarette use was common among youth.^1,2^ Among youth who currently use e-cigarettes, ∼83% reported flavored e-cigarette use in 2020.^3^ Use of flavors among youth was also commonly reported for other tobacco products.^2^ In 2019, among youth who currently use cigarettes and cigars, 47% reported smoking menthol cigarettes and 42% reported flavored cigar use, respectively.^2^

Most tobacco use starts in adolescence. Health risks of youth combustible tobacco product use are well known; e-cigarette aerosol, while generally containing fewer harmful chemicals than cigarettes, can contain nicotine, heavy metals like lead, volatile organic compounds, and cancer-causing agents.^4^ Nicotine is highly addictive and can harm adolescent and young adult brain development and may lead to the use of other tobacco products, including cigarettes.^4^

Flavors are known to promote youth tobacco experimentation, initiation, and polytobacco use.^5–8^ Most current research on flavors has focused on menthol; menthol cigarette smokers initiate smoking at younger ages and report less adverse reactions (e.g., nausea, difficulty inhaling) to their first cigarette than nonmenthol cigarette smokers.^9,10^ Users of flavored tobacco products perceive these products to be less harmful than nonflavored products^6^ and there is some evidence that perceived ease of use of flavored tobacco products may vary by race and ethnicity. Chen-Sankey et al analyzed Population Assessment of Tobacco and Health (PATH) study data and found that non-Hispanic (NH) Black and Hispanic youth perceived flavored products as easier to use than NH white youth.^5^ Furthermore, youth who believed flavored products were easier to use were more likely to report later use.^5^

Currently, the only flavor available in cigarettes is menthol. The 2009 Family Smoking Prevention and Tobacco Control Act, under FDA authority, banned the inclusion of characterizing flavors other than tobacco and menthol in cigarettes.^11^ Following the passage of this act, a reduction in cigarette use among youth was observed over the next 4 years.^5,12^ For e-cigarettes, JUUL, makers of the most commonly used e-cigarette in the U.S. market at the time these data were collected,^13,14^ voluntarily removed certain flavored (mango, creme, fruit, and cucumber) cartridges from retail stores beginning in November 2018 and from online stores beginning in October 2019; JUUL removed mint-flavored cartridges entirely from the market in November 2019.^15^

On February 6, 2020, the FDA implemented a policy prioritizing enforcement against the manufacture, distribution, and sale of certain unauthorized flavored prefilled pod or cartridge-based e-cigarettes (excluding tobacco or menthol).^16^ However, certain flavored e-cigarettes, including flavored disposable products, are still marketed and sold, as are menthol cigarettes, flavored cigars, hookah/shisha, and smokeless tobacco products.^17^

Tobacco product harm perceptions are a negative predictor of youth tobacco use^18^ and harm perception is associated with cessation metrics.^19^ In a longitudinal analysis of PATH data, youth who perceived e-cigarettes, hookah, and smokeless tobacco as “less harmful” than cigarettes at wave one were more likely to be new users of those tobacco products at wave two.^20^ Lower tobacco product harm perceptions were also associated with lower tobacco quit intentions and attempts.^21^

While perceptions that intermittent use of e-cigarettes had “no” or “little” harm decreased among youth during 2016–2020, in 2020, 20% of youth still endorsed the misperception that e-cigarettes had no or little harm. Concerningly, the belief that cigarettes had “no” or “little” harm increased among youth during 2016–2020.^21^ In a longitudinal study, e-cigarette susceptibility predicted future e-cigarette initiation and current use. e-Cigarette susceptibility among youth increased from 2014 to 2016 and decreased from 2016 to 2018 and differences in susceptibility by race and harm perceptions were observed.^22^

Those in various racial and ethnic groups can disproportionately experience inequitable structural practices and social conditions, discrimination, poverty, and stress, which can increase commercial tobacco use.^23^ Racial and ethnic groups have experienced a greater disease burden from tobacco use and have been subject to targeted marketing strategies from tobacco companies, including online marketing, and a higher prevalence and deals and coupons in minority neighborhoods.^24–27^ Targeted marketing and promotion of tobacco products and flavors are among the most studied inequitable structural conditions and social practices related to tobacco product use, and may help explain why cigarette smoking prevalence and tobacco product use preferences have historically differed among racial and ethnic groups.^28^

There is evidence that exposure to this targeted marketing may increase curiosity and susceptibility to tobacco products among youth in general, and racial and ethnic minority populations may have differing susceptibilities to this effect.^29,30^ Longitudinal studies have revealed that youth reporting higher initial receipt of tobacco coupons and more online tobacco marketing engagement later report higher rates of past 30-day use of cigarettes, e-cigarettes, cigars, smokeless tobacco, and hookah, suggesting a direct influence of marketing on eventual behavior.^31^

There are limited studies that characterize differences in youth flavored tobacco product use prevalence, curiosity/susceptibility, and harm perceptions by race/ethnicity. Given the disproportionate marketing, promotion, and use of flavored tobacco by racial and ethnic minority populations, it is important to understand differences in flavored tobacco product use and harm perceptions within these groups and to establish baseline data in light of rapidly changing regulations on flavored tobacco products. Using National Youth Tobacco Survey (NYTS) 2019 and 2020 data, this study takes a closer look at flavored tobacco product use, tobacco harm perceptions, and curiosity and susceptibility toward tobacco products among different racial and ethnic population groups.

## Methods

### Survey methods

Data came from the 2019 and 2020 NYTS, annual cross-sectional surveys administered electronically to students in grades 6–12 attending public and private schools. NYTS uses a three-stage cluster sampling design to provide a nationally representative sample. In 2019, 19,018 students completed surveys, and in 2020, 14,531 students completed surveys. For the 2020 NYTS, data collection was ended early due to widespread school closures as a result of the coronavirus disease 2019 (COVID-19) pandemic, resulting in a smaller, yet still representative sample size.

The overall response rates were 66.3% in 2019 and 43.6% in 2020. A more detailed description of NYTS methodology is available on the NYTS website.^32^ Racial/ethnic groups of interest included NH White, NH Black, Hispanic, or NH Other youth. Limited sample size prevented a more detailed exploration of differences within these groups. As a secondary analysis of deidentified public use data, this study was exempt from human subject review.

### Past 30-day tobacco product use measures

Past 30-day use of e-cigarettes, cigarettes, cigars (cigars, little cigars, and cigarillos), smokeless tobacco (chewing tobacco, snuff, dip, snus, and dissolvable tobacco), hookahs, pipe tobacco, bidis (small brown cigarettes wrapped in a leaf), and heated tobacco products was assessed. Past 30-day use was defined as using a respective product on ≥1 day during the past 30 days. Any tobacco product use was defined as past 30-day use of one or more of the eight assessed tobacco products. Any combustible tobacco product use was defined as past 30-day use of one or more of the following products: cigarettes, cigars, hookahs, pipe tobacco, and bidis. Noncigarette combustible tobacco use was defined as past 30-day use of one or more of the following: cigars, hookahs, pipe tobacco, and bidis, and not currently using cigarettes. Polytobacco use was defined as past 30-day use of two or more of the eight tobacco products mentioned above.

### Past 30-day flavored tobacco product use measures

#### Any flavored tobacco product user and flavored e-cigarette user

In 2019, among students who reported past 30-day use of any tobacco product, flavored tobacco product use was determined by the question, “Which of the following tobacco products that you used in the past 30 days were flavored to taste like menthol (mint), alcohol (wine or cognac), candy, fruit, chocolate, or any other flavors?” Participants could select from a list of options to indicate the flavored tobacco product or products they had used. Among students who reported past 30-day use of each respective product, those who selected the flavored product were categorized as a flavored product user.

On the 2020 survey, a separate question was asked among participants who currently used each tobacco product: “Were any of the [tobacco product type] that you used in the past 30 days flavored to taste like menthol, mint, clove or spice, alcohol (wine, cognac), candy, fruit, chocolate, or any other flavor?” Participants who responded “yes” for any of the seven tobacco products listed above were categorized as a flavored product user. For both years, participants who selected that they had used a flavored e-cigarette were considered to be a flavored e-cigarette user. Because of differences in the questions between 2019 and 2020, we were not able to study specific flavors.

#### Menthol cigarettes

In 2019 and 2020, menthol cigarette smoking was ascertained among past 30-day cigarette smokers from responses to two questions: (1) “During the past 30 days, were the cigarettes that you usually smoked menthol?” and (2) “During the past 30 days, what brand of cigarettes did you usually smoke?” Those reporting “yes” to the menthol question or who reported “Newport” or “Kool” as the usual cigarette brand were categorized as flavored (menthol) cigarette smokers.

#### Other flavored tobacco product user

Participants who selected that they used flavored versions of any of the other six tobacco products studied (cigars, smokeless tobacco, hookahs, pipe tobacco, bidis, and heated tobacco products) in the past 30 days were categorized as other flavored tobacco product users.

#### Susceptibility and curiosity

Curiosity and susceptibility were assessed among participants who had never used each respective tobacco product. As the 2019 and 2020 NYTS both included questions to assess curiosity about and susceptibility to e-cigarettes, cigarettes, and cigars, data for these 2 years were combined for analysis. For curiosity, respondents were asked, “Have you ever been curious about (using an e-cigarette; smoking a cigarette; smoking a cigar, cigarillo, or little cigar)?” To capture any level of curiosity, responses were dichotomized as curious (definitely yes, probably yes, or probably not) and not curious (definitely not).^33^

For each tobacco product, three questions assessed susceptibility: (1) “Do you think that you will try (an e-cigarette; a cigarette; a cigar, cigarillo, or little cigar) soon?”; (2) “Do you think you will (use an e-cigarette; smoke a cigarette; smoke a cigar, cigarillo, or little cigar) in the next year?”; and (3) “If one of your best friends were to offer you (an e-cigarette; cigarette; cigar, cigarillo, or little cigar), would you (use; smoke; or try) it?” Consistent with previous literature,^34^ to differentiate between committed “never” tobacco product users from susceptible tobacco product users, susceptibility for each product was defined as a response other than “definitely not” to any of the three susceptibility questions or the curiosity question.

#### Harm perceptions

The 2019 and 2020 NYTS included questions to assess harm perceptions of intermittent use of the following tobacco products: e-cigarettes, cigarettes, smokeless tobacco (chewing, snuff, dip, or snus), and hookahs. All respondents were asked, “How much do you think people harm themselves when they (smoke cigarettes; use chewing tobacco, snuff, dip, or snus; use e-cigarettes; or smoke tobacco in a hookah or water pipe) some days but not every day?” Response options included “no harm,” “little harm,” “some harm,” and “a lot of harm.” The 2019 and 2020 NYTS included a question to assess harm perceptions of tobacco products: “How strongly do you agree with the statement ‘All tobacco products are dangerous’?” Response options included “strongly agree,” “agree,” “disagree,” and “strongly disagree.”

### Statistical analysis

Statistical analyses were conducted using SAS-callable SUDAAN software (version 11.0.3; RTI International) to account for the complex sampling design. Weighted prevalence estimates and 95% confidence intervals were calculated for all measures. All estimates are reported by self-reported race and ethnicity (NH White, NH Black, Hispanic, or NH Other). Estimates of tobacco product use were conducted among the entire study population. Analyses of flavor use were conducted among past 30-day tobacco product users. *t*-Tests were used to assess differences in prevalence between 2019 and 2020 and differences between racial/ethnic groups when compared to White NH students; a two-sided *p*-value <0.05 was considered statistically significant. Curiosity and susceptibility analyses were conducted among participants who had never used each respective tobacco product. Results with unweighted denominators <50 are not shown.

## Results

In 2019, 56.2% of participants were NH White, 13.3% were NH Black, 25.0% were Hispanic, and 5.5% were NH Other race. In 2020, 53.3% of participants were NH White, 12.4% were NH Black, 26.5% were Hispanic, and 7.8% were NH Other. In 2019, 47.7% of participants were female, and in 2020, ∼49.4% were female. The proportion of middle school students (grades 6–8) was 43.9% in 2019 and 43.7% in 2020.

### Past 30-day tobacco product use

In 2020, any past 30-day tobacco product use ranged from 17.8% (15.4–20.3%) among NH White students to 10.1% (6.9–14.6%) among NH Other students (Table 1). From 2019 to 2020, any tobacco product use declined for all racial groups. Use of combustible tobacco declined significantly for NH White students and NH Black students, but not for Hispanic students. For e-cigarettes, reported use among all racial groups significantly declined, with NH White and NH Black students showing the largest percent decline. Modest, but significant declines were reported in the “other combustible tobacco” category for NH White and NH Black students, but not Hispanic students. There were significant declines in polytobacco product use for NH white and NH black students, but not for Hispanic students.

**Table 1.** Weighted Prevalence Estimates of Middle and High School Students Who Reported Past 30-Day Tobacco Product Use by Product and Race/Ethnicity—National Youth Tobacco Survey, United States, 2019 and 2020

	NH White			NH Black			Hispanic^a^			NH Other		
	2019	2020	Difference 2020 vs. 2019 (95% CI)	2019	2020	Difference 2020 vs. 2019 (95% CI)	2019	2020	Difference 2020 vs. 2019 (95% CI)	2019	2020	Difference 2020 vs. 2019 (95% CI)
	% (95% CI)	% (95% CI)		% (95% CI)	% (95% CI)		% (95% CI)	% (95% CI)		% (95% CI)	% (95% CI)	
Any tobacco product use^b^	25.4 (23.3 to 27.7)	17.8 (15.4 to 20.3)	−7.7 (−11.1 to −4.2)^c^	20.0 (17.4 to 22.9)^d^	13.2 (11.3 to 15.4)^d^	−6.8 (−10.2 to −3.4)^c^	22.2 (20.4 to 24.2)^d^	17.2 (14.3 to 20.4)	−5.1 (−8.7 to −1.5)^c^	15.8 (12.7 to 19.4)^d^	10.1 (6.9 to 14.6)^d^	−5.6 (−10.7 to −0.6)^c^
Combustible tobacco^e^	8.5 (7.2 to 10.0)	5.9 (4.7 to 7.4)	−2.6 (−4.6 to −0.5)^c^	12.0 (10.1 to 14.3)^d^	9.2 (7.8 to 10.7)^d^	−2.9 (−5.6 to −0.2)^c^	8.8 (7.7 to 9.9)	8.1 (6.4 to 10.3)^d^	−0.6 (−2.9 to 1.6)	5.5 (3.9 to 7.8)^d^	—	—
Cigarettes	5.0 (3.9 to 6.4)	3.7 (2.8 to 4.8)	−1.3 (−3.0 to 0.3)	3.1 (2.3 to 4.1)^d^	—	—	3.6 (2.8 to 4.5)^d^	3.6 (2.6 to 4.9)	0.0 (−1.4 to 1.4)	—	—	—
Other combustible tobacco^f^	6.2 (5.3 to 7.3)	3.8 (3.0 to 4.9)	−2.4 (−3.9 to −0.9)^c^	10.8 (8.9 to 13.0)^d^	8.0 (6.8 to 9.5)^d^	−2.7 (−5.4 to −0.1)^c^	7.4 (6.4 to 8.7)	6.6 (5.0 to 8.5)^d^	−0.9 (−3.0 to 1.2)	3.7 (2.5 to 5.6)^d^	—	—
e-Cigarettes	23.1 (21.1 to 25.1)	15.5 (13.5 to 17.8)	−7.5 (−10.6 to −4.5)^c^	13.6 (11.5 to 16.1)^d^	6.2 (4.8 to 8.1)^d^	−7.4 (−10.3 to −4.5)^c^	18.7 (16.9 to 20.7)^d^	13.7 (11.0 to 16.9)	−5.0 (−8.5 to −1.5)^c^	13.6 (10.9 to 16.9)^d^	7.7 (5.0 to 11.8)^d^	−5.9 (−10.3 to −1.5)^c^
Smokeless tobacco^g^	4.5 (3.4 to 6.0)	3.0 (2.3 to 3.9)	−1.5 (−3.1 to 0.1)	—	—	—	2.4 (1.9 to 3.0)^d^	1.7 (1.3 to 2.2)^d^	−0.7 (−1.4 to 0.0)	—	—	—
Polytobacco use^h^	8.9 (7.5 to 10.5)	6.1 (4.9 to 7.6)	−2.7 (−4.9 to −0.6)^c^	8.4 (6.8 to 10.4)	4.9 (3.9 to 6.0)	−3.6 (−5.7 to −1.4)^c^	7.6 (6.7 to 8.6)	6.7 (5.1 to 8.7)	−0.9 (−2.9 to 1.1)	5.1 (3.8 to 6.8)^d^	—	—

—, Results suppressed due to denominator <50.

^a^ Hispanic persons could be of any race.

^b^ Any tobacco product included e-cigarettes, cigarettes, cigars (cigars, little cigars, and cigarillos), smokeless tobacco (chewing tobacco, snuff, dip, snus, and dissolvable tobacco), hookahs, pipe tobacco, bidis, and heated tobacco products.

^c^ Represents significant difference in prevalence (*t*-test *p*<0.05) between 2019 and 2020.

^d^ Represents significant difference in prevalence (*t*-test *p*<0.05) compared against White NH youth from same year.

^e^ Combustible tobacco was defined as the use of cigarettes, cigars, pipes, bidis, or hookahs.

^f^ Other combustible tobacco was defined as the use of cigars, pipes, bidis, or hookahs.

^g^ Smokeless tobacco was defined as the use of chewing tobacco, snuff, dip, snus, or dissolvable tobacco products.

^h^ Polytobacco use was defined as using two or more tobacco products.

CI, confidence interval; e-cigarette, electronic cigarette; NH, non-Hispanic.

### Past 30-day flavored tobacco product use

In 2020, more than half (56.4–82.6%) of students who reported past 30-day tobacco use in each racial group reported use of any flavored tobacco product (Table 2). Past 30-day use of any flavored tobacco product ranged from 82.6% (80.4–84.6%) among NH white students to 56.4% (46.9–65.4%) among NH Black students (78.2%, 63.1–88.3%). From 2019 to 2020, use of any flavored tobacco product increased for all products across racial groups, except for menthol cigarettes. The lowest prevalence of any flavored tobacco product use was reported by NH Black students across most products in both 2019 and 2020; however, use of flavored e-cigarettes in this group increased from 43.9% in 2019 to 71.5% in 2020. Use of other flavored tobacco products increased significantly for all groups, except NH students of other races, with Hispanic students reporting the largest percent increase (30.3%).

**Table 2.** Weighted Prevalence Estimates of Middle and High School Students Who Reported Past 30-Day Flavored Tobacco Product Use Among Those Who Reported Past 30-Day Tobacco Product Use, by Product and Race/Ethnicity—National Youth Tobacco Survey, United States, 2019 and 2020

	NH White			NH Black			Hispanic^a^			NH Other		
	2019	2020	Difference 2020 vs. 2019 (95% CI)	2019	2020	Difference 2020 vs. 2019 (95% CI)	2019	2020	Difference 2020 vs. 2019 (95% CI)	2019	2020	Difference 2020 vs. 2019 (95% CI)
	% (95% CI)	% (95% CI)		% (95% CI)	% (95% CI)		% (95% CI)	% (95% CI)		% (95% CI)	% (95% CI)	
Any flavored tobacco product^b^	76.2 (74.0 to 78.2)	82.6 (80.4 to 84.6)	6.4 (3.6 to 9.3)^c^	48.2 (42.6 to 53.8)^d^	56.4 (46.9 to 65.4)^d^	8.2 (−2.4 to 18.9)	62.7 (58.8 to 66.5)^d^	75.0 (71.2 to 78.5)^d^	12.3 (6.9 to 17.7)^c^	67.0 (57.0 to 75.8)	78.2 (63.1 to 88.3)	11.2 (−4.2 to 26.5)
Flavored e-cigarette	75.0 (72.4 to 77.5)	85.9 (83.3 to 88.1)	10.8 (7.4 to 14.3)^c^	43.9 (37.3 to 50.8)^d^	71.5 (57.4 to 82.4)^d^	27.6 (13.3 to 42.0)^c^	63.0 (58.5 to 67.2)^d^	78.2 (74.1 to 81.8)^d^	15.2 (9.2 to 21.1)^c^	66.8 (57.0 to 75.4)	86.4 (70.8 to 94.3)	19.6 (5.6 to 33.6)^c^
Menthol cigarettes	46.4 (40.8 to 52.1)	38.2 (28.6 to 48.9)	−8.2 (−19.8 to 3.4)	—	—	—	50.8 (42.7 to 58.8)	38.8 (27.2 to 52.0)	−11.9 (−26.9 to 3.0)	—	—	—
Other flavored tobacco product^e^	49.6 (44.2 to 55.1)	63.2 (57.5 to 68.6)	13.6 (5.9 to 21.2)^c^	37.5 (31.7 to 43.7)^d^	45.1 (34.8 to 55.9)^d^	7.6 (−4.7 to 19.9)	37.2 (30.7 to 44.1)^d^	67.4 (61.6 to 72.8)	30.3 (21.8 to 38.7)^c^	—	—	—

—, Results suppressed due to denominator <50.

^a^ Hispanic persons could be of any race.

^b^ Any flavored tobacco product included e-cigarettes, cigarettes, cigars (cigars, little cigars, and cigarillos), smokeless tobacco (chewing tobacco, snuff, dip, snus, and dissolvable tobacco), hookahs, pipe tobacco, bidis, and heated tobacco products.

^c^ Represents significant difference in prevalence (*t*-test *p*<0.05) between 2019 and 2020.

^d^ Represents significant difference in prevalence (*t*-test *p*<0.05) compared against White NH youth from same year.

^e^ Other flavored tobacco product included cigars (cigars, little cigars, and cigarillos), smokeless tobacco (chewing tobacco, snuff, dip, snus, and dissolvable tobacco), hookahs, pipe tobacco, bidis, and heated tobacco products.

### Curiosity and susceptibility

Among students who had never used specific tobacco products included in this analysis (e-cigarette, cigarette, cigar, cigarillo, or little cigar), any product curiosity was the highest for e-cigarettes (5.0–6.0%) (Table 3). Susceptibility was also the highest for e-cigarettes (31.8–42.3%) compared to cigarettes or cigars, and this pattern was consistent across all racial groups. The group with the highest susceptibility to future e-cigarette use was Hispanic students (42.3%). Hispanic students had the highest susceptibility to future use of cigarettes and cigars as well. NH Black and Hispanic students were more curious about cigarettes; and NH Blacks were more curious about cigars than NH white students.

**Table 3.** Number and Percentage of Middle and High School Students Who Reported Curiosity About and Susceptibility to Tobacco Product Use Among Never Users of Each Specific Product by Race/Ethnicity—National Youth Tobacco Survey, United States, 2019 and 2020 Combined

	NH White		NH Black		Hispanic^a^		NH Other	
	***N***	% (95% CI)	***N***	% (95% CI)	***N***	% (95% CI)	***N***	% (95% CI)
e-Cigarettes								
Curious	597	5.3 (4.8 to 5.9)	182	5.8 (4.8 to 7.1)	411	6.0 (5.3 to 6.8)	89	5.0 (3.8 to 6.6)
Susceptible	3903	36.0 (34.6 to 37.3)	1011	31.8 (29.5 to 34.1)^b^	2868	42.3 (40.5 to 44.1)^b^	617	34.4 (30.7 to 38.4)
Cigarettes								
Curious	473	3.0 (2.6 to 3.4)	155	4.3 (3.3 to 5.6)^b^	392	4.5 (3.9 to 5.2)^b^	88	3.4 (2.5 to 4.5)
Susceptible	3612	24.7 (23.2 to 26.3)	748	19.2 (17.0 to 21.6)^b^	2719	30.5 (28.5 to 32.5)^b^	528	23.9 (20.8 to 27.2)
Cigars								
Curious	860	2.7 (2.4 to 3.1)	394	3.8 (3.0 to 4.8)^b^	136	3.4 (2.9 to 4.0)	277	2.4 (1.6 to 3.7)
Susceptible	3903	26.4 (25.2 to 27.7)	892	26.1 (24.1 to 28.2)	2835	31.9 (30.5 to 33.5)^b^	509	24.7 (21.2 to 28.5)

Unweighted number of students. Weighted column prevalence estimates. Assessed by the question, “Have you ever been curious about (tobacco product)?” Responses were recoded as highly curious (definitely yes, probably yes, or probably not) and not curious (definitely not). Assessed by the questions, “Do you think that you will use (tobacco product) soon? Do you think you will use (tobacco product) in the next year? If one of your best friends were to offer you (tobacco product), would you try it? Have you ever been curious about (tobacco product)?” Susceptibility was defined as a response other than “definitely not” to any of the four questions.

^a^ Hispanic persons could be of any race.

^b^ Represents significant difference in prevalence (*t*-test *p*<0.05) compared against White NH youth.

### Harm perception

Slightly more than half the respondents (51.7%) perceived “a lot of” harm could be caused by intermittent cigarette use, and this general pattern was noted among all racial and ethnic groups: NH White (50.8%), NH Black (59.9%), Hispanic (49.6%), and NH students of other races (51.0%) (Table 4). Regarding intermittent e-cigarette use, across all racial groups, more than 70% believed they caused “some harm” or “a lot of harm,” but there was some variation across racial groups. Over 40% of NH Black (41.3%) and NH Other (42.3%) students perceived intermittent e-cigarette use as causing “a lot of harm.” NH Black students (59.9%) also had the highest prevalence of perceiving intermittent cigarette use as causing “a lot of harm.”

**Table 4.** Harm Perceptions of Middle and High School Students by Race/Ethnicity—National Youth Tobacco Survey, United States, 2019 and 2020

	NH White		NH Black		Hispanic^a^		NH Other	
	***N*** ^b^	%^c^ (95% CI)	***N***	% (95% CI)	***N***	% (95% CI)	***N***	% (95% CI)
How much do you think people harm themselves when they smoke cigarettes some days, but not every day								
No harm	269	1.6 (1.4 to 2.0)	170	4.1 (3.0 to 5.5)	326	3.6 (3.1 to 4.2)	56	2.2 (1.4 to 3.3)
Little harm	1201	7.7 (7.1 to 8.4)	258	6.2 (5.2 to 7.3)	874	9.3 (8.5 to 10.1)	128	5.3 (4.2 to 6.7)
Some harm	6457	39.9 (38.7 to 41.1)	1202	29.9 (28.1 to 31.7)	3655	37.5 (35.9 to 39.2)	920	41.5 (38.5 to 44.4)
A lot of harm	8378	50.8 (49.4 to 52.1)	2345	59.9 (57.3 to 62.5)	4901	49.6 (47.7 to 51.5)	1249	51.0 (48.0 to 54.0)
How much do you think people harm themselves when they use e-cigarettes some days, but not every day?								
No harm	714	4.4 (3.8 to 5.2)	275	6.6 (5.5 to 7.8)	581	6.3 (5.5 to 7.1)	94	3.9 (2.8 to 5.5)
Little harm	3196	19.5 (18.4 to 20.6)	719	17.7 (16.2 to 19.3)	1928	19.8 (18.4 to 21.3)	339	15.6 (12.9 to 18.7)
Some harm	6612	40.7 (39.5 to 41.8)	1327	34.5 (32.5 to 36.5)	3852	39.4 (37.8 to 41.0)	920	38.1 (35.2 to 41.1)
A lot of harm	5748	35.5 (33.9 to 37.0)	1616	41.3 (39.0 to 43.6)	3339	34.6 (33.1 to 36.1)	993	42.3 (37.7 to 47.2)
How much do you think people harm themselves when they use chewing tobacco, snuff, dip, snus, or dissolvable tobacco products some days, but not every day?								
No harm	377	2.3 (2.0 to 2.7)	158	3.8 (3.0 to 4.8)	335	3.8 (3.2 to 4.4)	53	2.1 (1.4 to 3.2)
Little harm	1643	10.4 (9.5 to 11.2)	343	8.3 (7.3 to 9.5)	868	9.5 (8.5 to 10.5)	144	6.8 (5.5 to 8.5)
Some harm	6391	39.4 (38.3 to 40.5)	1078	27.7 (26.0 to 29.4)	3463	35.3 (34.1 to 36.6)	830	34.9 (32.1 to 37.8)
A lot of harm	7860	47.9 (46.4 to 49.4)	2381	60.2 (58.2 to 62.2)	5044	51.4 (49.7 to 53.2)	1316	56.1 (52.7 to 59.5)
How much do you think people harm themselves when they smoke tobacco in a hookah or waterpipe some days, but not every day?								
No harm	433	2.8 (2.4 to 3.2)	277	6.4 (5.3 to 7.8)	460	5.0 (4.3 to 5.7)	79	3.4 (2.4 to 4.9)
Little harm	1922	11.9 (11.1 to 12.7)	630	16.6 (15.0 to 18.3)	1340	14.3 (13.2 to 15.5)	257	11.6 (9.4 to 14.1)
Some harm	6753	42.1 (41.1 to 43.1)	1261	32.2 (30.2 to 34.2)	3755	38.6 (37.3 to 39.9)	897	39.9 (37.1 to 42.7)
A lot of harm	7099	43.2 (41.9 to 44.5)	1766	44.8 (42.6 to 47.0)	4109	42.1 (40.6 to 43.7)	1096	45.1 (40.9 to 49.4)
How strongly do you agree with the statement, ‘All tobacco products are dangerous’?								
Strongly agree	8361	51.3 (49.7 to 52.9)	2016	50.1 (47.8 to 52.3)	4614	47.2 (45.6 to 48.9)	1340	57.5 (53.6 to 61.3)
Agree	6068	37.5 (36.3 to 38.8)	1288	33.7 (31.7 to 35.8)	3729	39.1 (37.8 to 40.5)	785	32.9 (29.9 to 36.1)
Disagree	1261	7.8 (7.1 to 8.5)	355	9.1 (8.0 to 10.3)	815	8.5 (7.8 to 9.3)	130	6.3 (4.8 to 8.1)
Strongly disagree	540	3.4 (3.0 to 3.9)	269	7.1 (6.1 to 8.3)	481	5.1 (4.6 to 5.7)	80	3.3 (2.5 to 4.4)

^a^ Hispanic persons could be of any race.

^b^ Unweighted number of students.

^c^ Weighted column prevalence estimates.

Perceiving “no harm” from any of the tobacco products was generally more prevalent among NH Black and Hispanic students. NH white (51.3%), NH Black (50.1%), Hispanic (47.2%), and students of other races (57.5%) strongly agreed with the statement “all tobacco products are dangerous.” Across all racial/ethnic groups, the perception that intermittent e-cigarette use causes “a lot of harm” appears to be less prevalent than intermittent use of all other products causing “a lot of harm.”

## Discussion

From 2019 to 2020, the use of nearly every tobacco product declined across all racial/ethnic groups, with some of the largest percent decreases in use among NH Black students. Among NH Black students, the flavored tobacco products most commonly used were in the “other” category, which includes cigars, pipes, bidis, and hookahs. These findings are consistent with other studies that have found high use of cigars among Black students.^2^ In contrast, there were few declines for Hispanic students between 2019 and 2020, with the only significant change reported in e-cigarette use (−5%). Hispanic students reported the second highest prevalence of other tobacco product use and past 30-day users reported a 30% increase in the use of other flavored tobacco product use.

Among past 30-day tobacco users, the use of flavored tobacco products significantly increased across NH White and Hispanic students. Large absolute percent increases were seen in flavored e-cigarette use for NH Black students (27.6%), Hispanic students (15.2%), and NH students of other races (19.6%). There could be several reasons for these observed increases; however, it is difficult to disentangle the effects of rapidly changing regulations on the use of tobacco products, especially e-cigarettes, and two significant public health emergencies (e-cigarette, or vaping, product use-associated lung injury [EVALI] and COVID-19) from the results reported in this study.

In December 2019, a federal law was passed to raise the minimum age of sale of tobacco products, including e-cigarettes, to 21 years. In August 2019, the EVALI outbreak was strongly linked to the use of THC in e-cigarettes^35^ [Outbreak of Lung Injury Associated with the Use of E-Cigarette, or Vaping, Products | Electronic Cigarettes | Smoking & Tobacco Use | CDC), which was followed by the COVID-19 pandemic (Coronavirus Disease 2019 (COVID-19) | CDC] in March 2020. In February 2020, FDA issued an enforcement policy prohibiting the sale of flavored prefilled cartridge e-cigarettes.

Sales data from 2020 found that market shares of disposable e-cigarettes (which could still contain flavors other than menthol in 2020) and menthol-flavored prefilled cartridges significantly increased in 2020, following the new federal policy.^36^ Increases in the use of flavored other tobacco products, especially among Hispanic youth, are concerning. Flavors improve the taste and reduce the harshness of tobacco products, making easier to become addicted to those products.^37^ Nonetheless, decreases in tobacco product use, especially menthol cigarette use, are encouraging and could lead to a reduction in future tobacco-related disease. More information is needed to see if NH Black youth have also experienced a reduction in menthol cigarette use in more recent years.

It is unknown what may be driving differential changes in flavored tobacco use across racial and ethnic groups, but it could be related to differences in tobacco curiosity and susceptibility. Among the entire sample, curiosity and susceptibility were highest regarding e-cigarettes compared to other tobacco products. While curiosity and susceptibility measures cannot predict all future tobacco use,^38^ they are important factors in assessing increased risk of youth becoming established tobacco product users.^33,39^ Regarding perceptions of harm, across all race/ethnicity groups, the vast majority of respondents viewed tobacco products, even when used intermittently, as having some or a lot of harm. The proportion of students strongly agreeing with the statement “all tobacco products are dangerous” was similarly high across all racial/ethnic groups. NH Black students had highest harm perception for cigarettes, which is noteworthy, considering mixed results in adult harm perceptions in other studies.

One study by Bernat et al found NH Black adults had similar harm perceptions of cigarettes compared to NH white adults,^40^ while another study found that cigarette harm perceptions in NH Black adults differed by menthol preference, with NH Black menthol adults who smoke perceiving their own brands as more harmful.^41^ Hispanic youth had the highest percent susceptibility and curiosity for e-cigarettes and cigarettes, along with the highest susceptibility for cigars. These findings combined with large percent differences in other flavored product use, as well as increases in marijuana vaping seen in other studies,^42^ indicate the urgent need for expanded research into Hispanic youth tobacco use to prevent a disproportionate burden of tobacco-related disease in this group.

There is scarce information about differences in flavored tobacco use and harm perceptions among racial and ethnic minority groups, especially Hispanic youth; therefore, possible explanations for our observed results are limited and detailed speculation would be unwarranted. There is evidence that racial and ethnic minority youth may be exposed to more online tobacco marketing as well as marketing administered through other platforms than NH white youth, which could increase susceptibly and curiosity.^25,30^ There is also evidence that Hispanic people have been targeted by the tobacco industry due to their high purchasing power and, thus, may be more susceptible than other groups to marketing efforts by the tobacco industry,^29^ which was reflected in our findings on susceptibility. Future research could focus on exploring reasons for these differences and explore how susceptibility, curiosity, and harm perception affect initiation of products, especially flavored tobacco products.

There are limitations to this investigation. Information was self-reported, and students were asked about socially undesirable behaviors; therefore, results are subject to recall and social desirability bias. Some of the cell sizes were small, limiting our ability to report on certain racial and ethnic groups, especially menthol cigarette use among NH Black youth. Changes between 2019 and 2020 in categories such as cigarettes (including menthol) and smokeless tobacco could not be assessed due to small cell sizes for some racial/ethnic groups and is a limitation of this study. There was a change in the way flavored tobacco product use was assessed, which meant that we were unable to examine flavored tobacco use for individual products other than menthol and comparability may be limited between years.

And finally, the data collection period was truncated because of the COVID-19 pandemic, resulting in a lower school participation rate (49.9%) compared with recent NYTS cycles. However, the 2020 NYTS student participation rate (87.4%) remained high. Despite these limitations, this study has several strengths. NYTS produces nationally representative estimates of tobacco use among youth in middle and high schools and the overall sample size is robust. This is the most recent and comprehensive examination of racial and ethnic differences in overall and flavored tobacco use prevalence and measure of curiosity, susceptibility, and harm perception to date.

For most groups examined, flavored tobacco product use increased among past 30-day users, and Hispanic youth are especially at risk. FDA continues to monitor and take action to prohibit some companies from selling youth-appealing, flavored disposable e-cigarettes and flavored e-liquids without authorization, while several states and localities have restricted flavored tobacco product sales, including sales of menthol-flavored products.^43^ These results indicate that racial/ethnic disparities in flavored tobacco use, harm perception, and susceptibility continue to exist. A better understanding of the social and environmental factors that drive inequities in tobacco use behaviors and perceptions, particularly for Hispanic youth, could lead to better targeted or tailored tobacco control interventions around flavored tobacco product use and susceptibility to all tobacco product use.

## Data Availability

NYTS data are publicly available and can be accessed at: https://www.cdc.gov/tobacco/data_statistics/surveys/nyts/index.htm

## Abbreviations Used

CI: confidence interval
COVID-19: coronavirus disease 2019
e-cigarette: electronic cigarette
EVALI: e-cigarette, or vaping, product use-associated lung injury
NH: non-Hispanic
NYTS: National Youth Tobacco Survey
PATH: Population Assessment of Tobacco and Health
